# Towards individualisation of treatment in endometrial cancer

**DOI:** 10.1038/bjc.2012.140

**Published:** 2012-05-08

**Authors:** S E Taylor, J A Green

**Affiliations:** 1 Department of Gynaecological Oncology, Liverpool Women's Hospital, Crown Steet, Liverpool L7 8SS, UK; 2 Department of Molecular and Clinical Cancer Medicine, University of Liverpool, Liverpool L69 3BX, UK

Endometrial cancer is the most common gynaecological cancer in the Western world, and its incidence is rising in most European countries, largely owing to increasing obesity. The majority are early stage, low-grade tumours associated with a favourable prognosis, and current classifications describe type 1 and 2 categories based primarily on morphological and molecular criteria. Hormone therapy has been shown to be effective predominantly in type 1 tumours and subgroups identified by classical receptor evaluation (Decruze and Green, 2007). Recent clinical interest has focused on identifying poor prognosis tumours and evaluating the benefit of adjuvant cytotoxic chemotherapy. This follows the demonstration of survival gain in advanced disease (Humber *et al*, 2007), but these therapies are toxic and not suitable for many elderly patients with concurrent medical problems. Activation of the P13K pathway has been observed in ∼30% of type 1 endometrial cancers and 20% of type 2 endometrial cancers, although mTOR-directed targeted therapy has shown only modest activity to date. Other PI3K pathway alterations leading to deregulated signalling include *PIK3CA* amplification or mutation and mutation in the *AKT* gene (Dedes *et al*, 2011). These observations provide interesting challenges for a personalised approach to the treatment of these tumours.

The paper by Krakstad *et al* (2012) in this issue of the *BJC* takes tissue biomarker studies a substantial step forward in endometrial cancer. The study was adequately powered with a primary set of 182 cases, validated in a separate set of 474 cases and further confirmed by RNA expression in 237 cases where fresh tissue was available, thus conforming to the REMARK guidelines. The authors evaluated the relative contributions to prognosis of the classical nuclear steroid receptor ER*α* and the novel G-protein-coupled oestrogen receptor (GPER) in primary endometrial cancer. ER*α* and ER*β* predominantly function through genomic signalling events, while GPER stimulates EGFR, ERK1/2 and PI3K through a non-genomic rapid signalling mechanism, leading to widespread effects on neuroendocrine, immune and reproductive functions.

Since the discovery of ER*β* in 1996, the field of oestrogen signalling has become increasingly complex. Oestrogen has many important functions both as a locally synthesised hormone in the reproductive organs and as a circulating transcription factor. The repertoire of functional oestrogen receptors now includes several splice variants, which modify the effect of the classical receptors and/or provide alternative routes to transcriptional activation (Taylor *et al*, 2010). Those with evidence for functionality include ER*β*2, which has a defective ligand-binding domain and inhibits ER*α*-mediated signalling, ER*α*Δ3,which has a partially absent DNA-binding domain and indirectly stimulates transcription of a number of genes, and ER*α*36 a truncated membrane-associated receptor, which initiates non-genomic oestrogen signalling (Taylor *et al*, 2010; Zhang *et al*, 2011). G-protein-coupled oestrogen receptor is a separately encoded, non-classical oestrogen receptor and there are likely to be others, as yet undiscovered. A multitude of interacting receptors, together with a host of compounds with oestrogenic activity and differing effects on these receptors, enables the subtle regulation of oestrogenic responses. This may contribute to pathological processes. Understanding this complexity is likely to prove important in oestrogen-sensitive tumour biology.

The key findings of the Krakstad paper are that low cytoplasmic GPER expression was a marker of poor prognosis, as was low ER*α* expression. Loss of GPER also conferred a poor prognosis when the analysis was restricted to the ER*α* positive and the endometrioid histology subgroups. The double-negative GPER and ER*α* subgroup had the worst prognosis, and the majority of metastases also showed loss of either GPER or ER*α* expression. The majority of the patients in this study were of low grade and endometrioid histology, and within this subgroup these biomarkers are clearly useful in identifying a poor prognostic category. No information is given on treatments given to these patients, and those studies where this is available have generally had small numbers of patients (Decruze and Green, 2007). The authors provided some evidence based on RNA profiles supporting the use of HDAC inhibitors in ER+/GPER− endometrial cancers. However, combinations either with conventional agents or additional targeted therapies are likely to be necessary to have a major impact on first-line therapy.

In a study of 24 uterine carcinosarcomas, Huang *et al* (2010) demonstrated a correlation between ER*β* and GPER, with higher expression in advanced stage disease. A further small study (Smith *et al*, 2007) showed increased GPER was an unfavourable prognostic factor, in keeping with studies in breast cancer. Clearly, confirmation is required from centres or networks with adequate numbers of patients across the spectrum of uterine cancers.

Ideally, biomarkers should be assessed in tissue from relapsed patients as recent studies in other tumour types have confirmed extensive molecular heterogeneity between primary tumours and metastases (Gerlinger *et al*, 2012). In endometrial cancer, several years may elapse between initial diagnosis and instigation of systemic therapy. Non-squamous gynaecological cancers are heterogeneous, and the pathogenesis of these cancers has recently been reviewed (Kurman and Shih, 2010). Molecular similarity between endometrioid tumours arising from the endometrium and from the ovary leads to intriguing possibilities for selective approaches to treatment based on mutation profiles, rather than presumed tissue of origin.

This paper proposes GPER as a biomarker in endometrial cancer, which shows promise for incorporation into clinical practice, although this is neither a rapid nor an inexpensive process. In the meantime assessment of the ER status should become routine in endometrial cancers, where the criteria established in breast cancer will suffice for the present. Increasing treatment options in endometrial cancer make accurate histopathological categorisation and molecular profiling essential, although predictive factors related to the EGFR/PI3K pathways have not been validated sufficiently for routine use.
